# Healthcare experiences of uninsured and under-insured American Indian women in the United States

**DOI:** 10.1186/s41256-022-00236-4

**Published:** 2022-02-11

**Authors:** Jessica L. Liddell, Jenn M. Lilly

**Affiliations:** 1 University of Montana School of Social Work, Jeannette Rankin Hall 004, 32 Campus Dr, Missoula, MT 59812 USA; 2 Fordham University Graduate School of Social Service, New York, NY USA

**Keywords:** American Indian, Healthcare, Women, Health insurance

## Abstract

**Background:**

Extensive health disparities exist for American Indian groups throughout the United States. Although insurance status is linked to important healthcare outcomes, this topic has infrequently been explored for American Indian tribes. For state-recognized tribes, who do not receive healthcare services through the Indian Health Service, this topic has yet to be explored. The purpose of this study is to explore how having limited access to health insurance (being uninsured or under-insured) impact American Indian women's healthcare experiences?.

**Methods:**

In partnership with a community advisory board, this study used a qualitative description approach to conduct thirty-one semi-structured life-course interviews with American Indian women who are members of a state-recognized tribe in the Gulf Coast (United States) to explore their Western healthcare experiences. Interview were conducted at community centers, participant homes, and other locations identified by participants. Interviews were transcribed verbatim and findings were analyzed in NVivo using conventional content analysis. Findings were presented at tribal council meetings and to participants for member checking.

**Results:**

Themes identified by participants included: (a) lack of insurance as a barrier to healthcare; (b) pre-paying for childbirth when uninsured; and (c) access to public health insurance coverage. Twenty-four women mentioned the role or importance of insurance in discussing their healthcare experiences, which was referenced a total of 59 times.

**Conclusion:**

These findings begin to fill an important gap in the literature about the health insurance experiences of American Indian tribal members. Not having insurance was an important concern for participants, particularly for elderly and pregnant tribal members. Not having insurance also kept tribal members from seeking healthcare services, and from getting needed prescriptions. In addition to promoting knowledge about, and expanding insurance options and enrollment, increased sovereignty and resources for state-recognized tribes is needed to address the health disparities experienced by American Indian groups.

## Background

Affording healthcare services is a significant barrier for American Indian peoples in maintaining health. As part of treaty agreements signed between the U.S. federal government and federal American Indian tribes, there exists a trust responsibility that requires the government to provide for the healthcare of American Indian populations [1]. This trust agreement stipulates that the Indian Health Service (IHS) agency, run through the U.S. Department of Health and Human Services, is responsible for providing comprehensive health services for federally recognized tribal members [2]. However, this trust responsibility is not upheld, as reflected by the extensive health disparities experienced by American Indian people, including disproportionate levels of chronic diseases such as diabetes, heart disease and cancer [3], compounded by insufficient healthcare services resources and healthcare discrimination [1, 3].

Though the IHS has often failed in providing high-quality, or even adequate, healthcare services, state-recognized tribal members do not receive even these resources and benefits [4–10]. Tribes that are state-recognized do not receive services from IHS and are thus reliant on other forms of healthcare access through private or public insurance. Yet most research on American Indian healthcare policy focuses on the provision of services through IHS. To our knowledge no studies have explored the topic of American Indian women’s health and insurance status—an important gap this research begins to fill by examining the experiences of American Indian women who are members of a state-recognized tribe in the Gulf South in accessing healthcare. To contribute to limited knowledge in this area, we investigate how American Indian women pay for healthcare, and the impact of being uninsured or underinsured on their healthcare experiences.

Scholars have noted that healthcare in the United States is viewed as a commodity, and not as a right, which puts American Indian communities at particular risk since they have already “purchased” their healthcare (through treaty agreements) and are unable to negotiate for better quality care [11]. As one American Indian woman stated, “If they don’t want to provide us healthcare, then why don’t they give the land back?” [11, p. 8]. However, as noted previously, non-federally recognized tribes are additionally put at a disadvantage [12] since, despite critiques of IHS’s inadequate and problematic provision of services, it is frequently one of the main, or only source of healthcare for many American Indian individuals, and is not available to state-recognized tribes [8, 9, 13].

Even those tribal members who are eligible to receive services from IHS are encouraged to have additional coverage through insurance, since IHS is frequently insufficient to meet all healthcare needs of tribal members and is not considered health insurance [14]. Around half of American Indian people have some form of private health insurance, while 43.3% relying on Medicaid, the United States’ managed insurance program for lower-income individuals, and around 15% are uninsured [2] (This number adds up to more than 100% since participants could report having both private and some form of publicly-funded healthcare). An additional concern is that currently over half of uninsured Native Americans live in states that have not pursued Medicaid expansion [15]. Paying for healthcare is an important barrier for American Indian peoples in accessing healthcare. Previous research has found that American Indian individuals often make decisions about their healthcare based on their perceptions about their ability to pay for their care, and not based on recommendations from their doctor, or their desire to receive services [16–18]. Access is further complicated for American Indian groups, who frequently reside in more rural areas, where healthcare options are already limited and spread out, and the number of providers who accept a participant’s particular form of insurance may be restricted [19].

Insurance status is associated with seeking prenatal care and maternal and infant health outcomes and well-being, driving up maternal mortality and morbidity disparities [20, 21]. Insurance gaps are an important contributor to health disparities for American Indian women [21] because Medicaid coverage for pregnant women expires 60 days after birth, while many pregnancy-related complications occur up to one year-post childbirth [22]. Costs of paying for maternal healthcare may also impact women’s reproductive decision-making, as research with non-American Indian women has shown that those with higher out-of-pocket childbirth costs were less likely to have subsequent children [23]. This has important implications for reproductive autonomy if women are making decisions based on their perceived ability to pay for childbirth and not on their own reproductive desires. Although health disparities among American Indian groups are well-documented, the role that insurance status plays in impacting access to healthcare has been infrequently explored, and to our knowledge, has never been examined among state-recognized tribes who do not receive healthcare from IHS.

## Methods

### Research Design

This study employed a qualitative descriptive methodology, which is frequently used in semi-structured interviews as a pragmatic, naturalistic investigative approach [24]. This methodology is considered a culturally-congruent approach to research with American Indian peoples [25], and was considered appropriate for this study because it uses low-level interpretation, maintains cultural nuances, and highlights participants’ voices [24]. A qualitative descriptive methodology emphasizes research participants’ voices and understandings, rather than abstract descriptions of experiences, which makes it especially useful for health-focused research seeking to produce findings that can inform interventions [24, 25]. Our overarching research question was “How does having limited access to health insurance (being uninsured or under-insured) impact American Indian women's healthcare experiences?".

### Setting and Participants

This research was conducted with members of one state-recognized, Gulf Coast tribe. We keep the identity of the tribe confidential in accordance with agreements made with the tribal council and in alignment with recommendations for conducting culturally sensitive research with American Indian groups [25]. There about 17,000 members of this tribe dispersed throughout the Gulf Coast region, which is characterized by waterways, wetlands, water management facilities, and oil production infrastructure upon which tribal members depend for cultural and economic resources. The area in which this tribe is located has experienced significant environmental changes, including land loss and hurricanes. Tribal members have historically experienced educational discrimination, forced relocation, and denial of tribal recognition at the federal level which limits access to resources and hinders political autonomy. Important cultural values within this tribe include self-sufficiency, generosity, family closeness, and advocating for others.

We used a purposive sampling strategy and snowball sampling methods to recruit and enroll participants who met inclusion criteria—adult females who were enrolled members of the tribe. Thirty-one women who identified as members of the tribe participated in semi-structured qualitative interviews for this research. Due to the considerable difficulties that tribal members have historically had in proving tribal membership [26], we did not require proof of enrolled tribal membership for participation.

Participants ranged in age from 18 to 71 years old (*M* = 51.71). Most women (87.1%) had completed a GED or high school degree. About half of participants (51.61%) had completed some form of educational training after high school. The majority of women reported having some form of health insurance coverage (93.54%) and having at least one child (83.87%). Participants reported having two to three children on average. Women reported having their first child at around the age of 20, on average.

### Interview guide development

We worked with a community advisory board (CAB) throughout the study, which was composed of two women who were tribal community members. The CAB helped in developing interview questions, assisted in ensuring that the research methods were appropriate and culturally relevant, and helped to recruit participants and disseminate study findings.

### Data Collection

Before commencing study proceedings, we received approval from the University’s Institutional Review Board and the tribal council’s internal review board [number and institution omitted for blind review]. The first author performed and digitally-recorded all interviews with participant consent. Interviews focused on women’s reproductive health experiences and included questions such as: “Can you tell me about a time you needed sexual/reproductive healthcare? How do you usually pay for healthcare? Are you able to see a doctor whenever you need to? Can you tell me about an experience using the nearest healthcare facility?” Interviews took place at participants’ homes or in tribal community buildings, depending on participants’ preferences. Interviews were conducted between October 2018 and February 2019 and ranged in length from 30 to 90 min (*M* = 66 min). As recommended by the CAB, participants received a $30 gift card to thank them for their time in being interviewed.

### Data Analysis

All interviews were transcribed verbatim, using NVivo software for data processing and analysis. We employed qualitative content analysis—a form of data analysis frequently used in qualitative descriptive research [27]—as our analytic strategy. In this approach, codes emerge directly from participants’ voices, and theory can inform results [27]. Following this approach, the first author (PI) listened to each recorded interview three times, and then began an inductive coding process working from transcripts to develop an initial list of broad codes and themes. These initial codes were then refined into discrete codes for the final coding scheme [24]. For example, during the initial round of analysis a theme of lack of insurance was identified. During later rounds of analysis this was divided into sub-themes of “lack of insurance during pregnancy” and “the need for public assistance programs.” The second author then independently reviewed the data and the two authors discussed and reached consensus regarding key themes.

To ensure this study was carried out rigorously, this research adhered to Milne and Oberle’s (2005) [27] strategies of: (a) flexible and systematic sampling; (b) encouraging participants to speak freely and openly; (c) creating accurate, verbatim transcripts; (d) using participants’ language and experiences to drive coding; and (e) centering context throughout the analysis. We also conducted member-checking, inviting all participants who agreed to be contacted after the study with a summary of findings for review and feedback. This summary of results was provided to each participant at least twice. The first author also shared findings through presentations at tribal council meetings and events.

## Results

This exploration of American Indian women’s experiences of accessing and paying for healthcare revealed three main themes in participants’ experiences: (a) lack of insurance as a barrier to healthcare; (b) pre-paying for childbirth when uninsured; and (c) access to public health insurance coverage. Twenty-four women mentioned the role or importance of insurance in discussing their healthcare experiences, which was referenced a total of 59 times. Please see Table 1 for a summary of result findings.

**Table 1:** Summary of study themes

Main themes	Sub-themes	Exemplar quotes
Lack of Insurance as a Barrier to Healthcare	Lack of insurance norm for elders growing up	“Momma always paid for her doctor visits [out of pocket] until she got older and stuff like that. ….healthcare was just practically unheard of. We pretty much suffered our consequences with illnesses and that sort of thing.” (30)
	Foregoing care because couldn’t afford to pay out of pocket	“They do the free care with the doctor but then I have to buy the medicine…I don't bother with medicine.” (6)
	Seasonal work not providing healthcare benefits	“If they don't have insurance…that's the first question the doctors ask for…You know, even making an appointment, you know, so yeah….That's the barrier.” (30)
	Stigma related to going to charity hospital	
	Even if visits are free, some patients are unable to afford medications	
Pre-Paying for Childbirth when Uninsured	Older women reflected back on not having insurance when they gave birth	“They told us how much the bill was going to be, and we paid it. ‘Just make sure you have it paid before the baby’s born’” (21)
	Extra cost if complications during childbirth	
Access to Public Health Insurance Coverage	Importance of public assistance to meet health needs	“I’m thankful for Medicaid because I wouldn’t have been able to afford it” (29)
	Need for education/training about forms of assistance and how to enroll	“I think that would be something good to have like a Medicaid and Medicare to let them know what's available for them and all. Because a lot of people in this community don't have insurance and they've never really had insurance, you know?” (25)
	Younger tribal members age out of coverage and are left uninsured	“She's 19. Yeah, she aged out. So…she no longer has insurance” (2)

### Lack of Insurance as a Barrier to Healthcare

Participants described many barriers that prevented tribal members from accessing needed healthcare, most notably a lack of health insurance coverage. One participant reflected that when she was growing up, not having health insurance seemed to be the norm within the community:

A lot of our Indian people…growing up…they didn't have, they couldn't afford insurance, health insurance…I didn't get, momma and them didn't have health insurance until… I [don’t] think ever, momma always paid for her doctor visits [out of pocket] until she got older and stuff like that. ….healthcare was just practically unheard of. We pretty much suffered our consequences with illnesses and that sort of thing. When we did come [go to the doctor]…the bills would start coming in on top of trying to keep food on the table, I think that was difficult for most of the Indian families…my dad was a good provider, but I remember all the time my mom saying, I have to pay this bill, I have to pay this hospital, or this doctor bill, and so on…I don't think it was a status at all, it was just our parents had a sense of responsibility, and that sense of responsibility meant, you pay your bills…I know my mom was a stickler for paying bills, especially hospital bills and doctor bills and that sort of thing (30).

As this participant describes, healthcare was often forgone or delayed in the community because tribal families couldn’t afford health insurance nor the high cost of care without it. This participant’s family made great efforts to remain financially responsible when it came to medical bills, although they struggled to afford healthcare costs in addition to other family needs. Another participant described not going to the doctor as a child because she did not have health insurance. When asked if being uninsured kept tribal members from seeking out healthcare, she responded: “Correct…Honestly, I can't tell you of a time that I visited a doctor in a doctor's office as a child…As an adult, yes. But not as a child (23)” As this participant emphasizes, not having access to health insurance often meant also not having access to medical professionals.

One participant expressed concern that many tribal members were uninsured because their work was seasonal, and that this was especially concerning because of the hard labor many participants performed on the job:

We have a lot of disabilities because our guys work hard and when they work on the boats, rowboats and so they have lower back problems or whatever. So that's a big...these guys, to be an oystermen, or fisherman is very, very hard work...And the body is not designed to do that forever...They don't get it. They don't have insurance....We see that a lot...no insurance. So, they don't get proper care. They don't get the therapy that they need, mentally and physically...or the right meds (15).

As this participant explained, performing strenuous, seasonal labor for companies that do not provide employer-sponsored health insurance takes a significant toll on tribal members’ health. Another participant further elaborated on the connection between seasonal work and a lack of health insurance:

A lot of the natives don't have insurance. They don't have insurance...they don't have it through their job. A lot of people down here they shrimp, on the water, and so it's not offered...the shrimp business is so seasonal. Sometimes they do good, sometimes they don't, so they don't tend to [be] getting insurances, you know, because there's going to come a time that they won't be able to afford the insurance. $30, $40 dollars and sometimes they don't even make that, so they just don't do it. They have to wait until they're older [when they qualify for Medicare] to be able to get care, you know, really good care, you know (21).

As this participant emphasized, many people cannot afford health insurance coverage, which often resulted in delaying healthcare until participants are old enough to qualify for Medicare. When asked what participants do when they get sick, she stated:

They have like a free hospital here, [name of hospital omitted]. It's free and they go there. So, if the people here, if we hear that they went to [hospital name], "Oh, it's because they don't have insurance." And so, everybody knows that they probably don't have insurance, and that's why they go there (21).

When asked if there was a stigma about that, she responded: ‘Yeah, yeah. There is.” One participant also described how the precarious nature of her husband’s employment caused them to delay healthcare when they were younger: “So we didn't…go to the doctor, so it wasn't until I was grown…Cause I wasn't married to a man who didn't keep a job. So, we never had any kind of insurance or anything like that (23).”

One participant felt that not having health insurance combined with limited options for free or affordable care act as significant barriers for tribal members:

A lot of people don't have health insurance... a lot of people don't even know that they can't even go to [name of previously free clinic]... because you can't do free care there anymore (19).

As this participant suggests, free clinics or charity hospitals that once provided a viable alternative for uninsured, low-income people have become increasingly limited in the community. Another participant also stated that tribal members who didn’t have health insurance would have to go to the local free hospital if they needed health services: “We would go to the, the local charity hospital…you didn't have to pay anything to go there…I mean it took you a while to get seen, but that's where we would go. If we had something (24).” The long wait time for care at this hospital speaks to the volume of patients in need of free care in the community. Another participant similarly referenced the long wait times those without insurance experienced if they had to go to the free hospital:

I mean, people that don't have a job or income, I don't know what they do. I mean, we do have [hospital name omitted] here, but that could take days, weeks, months, sometimes to see a doctor. I applied for Medicaid, because I'm diabetic I got it, so that was a saving grace for me. But then you have to find a doctor who accepts that, because not all doctors accept it. So that was hard for me. In that two years, almost, that I didn't have health insurance, and I didn't have a job…So that's a big thing. I don't know what we can do to help them...you've got to have healthcare. I just wish you could go somewhere whether you had insurance or not and you could be taken care of the same as you and I go to the same place as this guy who needs meds for his whatever issues he's having...You have to wait so long (9).

For many uninsured community members, access to free or affordable healthcare was limited, requiring lengthy wait times, which can be particularly challenging for those with urgent or chronic conditions, or for those who may have ambivalent feelings about going to see a healthcare provider in the first place. In some cases, community members felt their best option was to access the emergency room when they needed medical attention, as one participant explained:

Especially if they can’t afford...their regular doctor or a... PCP [primary care physician] or they don't even have, a PCP…a lot of our Indian people…I ask them, “so who's your PCP?” And “I don't have one.”...I was like, “Okay, let's start this, you know, you're going to get it, we're going to get you to a PCP,” you know, get, and we have to use the state hospital...Especially if they can't afford it...let's get that established. And that could be two months down the line that they actually get in there, to be able to see them…So that's why...they use the emergency room...[as] their primary...Avenue. (30).

As these statements show, when one doesn’t have insurance that allows for preventive and routine care with a primary care physician, tribal members turned to the emergency room as their only viable opportunity to receive the care they needed in the time they needed.

Participants also noted that costs can be prohibitive, which limits their options for treatment when they need it. One participant identified further barriers to needed medical care for uninsured, low-income tribal members. She stated that she currently did not have insurance: “I don't have none (6).” Another participant described going to the free walk-in clinic if she needed care: “I haven't been to the doctor in a while. I don't really have any healthcare right now. I just go to the walk-in clinic for something (8).” They went on to describe that not being insured kept her from seeking needed medical care:

I need to go to the doctor....paying for all these tests to be done and paying for just these things. I know I need to do it...I know I need to save up before because all that stuff is so expensive. I don't know. Maybe I need to look in to see if there is healthcare for ... I guess there is for college students somewhere maybe. I don't know. I'm not sure (8).

Without health insurance coverage, the costs of medical care and continuing treatment are prohibitive, leaving low-income, uninsured community members little choice but to risk their health by foregoing or delaying healthcare or limiting their engagement with treatment.

### Pre-Paying for Childbirth when Uninsured

In discussing how they managed to pay for sexual and reproductive healthcare, older participants frequently described the norm that patients pre-pay for their delivery before childbirth if they didn’t have insurance. This was less common for younger participants, who were more frequently able to utilize public assistance during pregnancy and labor if they did not have private insurance. One participant described this experience with her first birth:

No, my first child, no, I had no insurance…. And he [her partner] was working and I was seeing a specialist to begin with because a friend of mine didn't have a specialist and she ended up having a [c] section and it costs them more money. So, he [partner] said, “Well, this is our first, let's go ahead and pay for a specialist. And...if we don't need it, we don't need it. But just in case.” …We had an x amount of dollars we have to put down, to reach, before they would deliver the baby, that they pay, did a payment plan for my daughter (15).

As this participant described, without insurance coverage, pre-paying was a requirement to deliver her baby at the local hospital. In situations such as this, paying for the delivery in installments becomes a feature of the pregnancy. Another participant also described the practice of paying the hospital for the delivery of the child before they were born:

We didn't have insurance at all. So, we had to pay ahead of time before the baby was born. We had to pay. And then I got pregnant and had to pay for another one, so I had like two bills for two babies. But we paid it off. We paid a little bit at a time, $50, $60 a month and so I paid both of them…We didn't have insurance...it was hard, but we did it, you know?...we're strong...We know what we need to do, you know, and we didn't try to get away from it (21).

This participant’s experience shows how it can be a struggle for low-income families without insurance to afford the costs associated with childbirth, especially for multiple children in a short time span. For this participant, being sure to pay off her debt to the hospital was something she saw as a sign of strength and responsibility. When asked what would have happened if she hadn’t been able to pay, she responded: “I don't know. Probably would have sent me to a free hospital maybe (21).”

Paying out of pocket in advance was common for pregnant women who were uninsured, though some were able to bear that financial responsibility more easily than others. One participant described pre-paying for childbirth when she didn’t have insurance, although when asked if it was hard to afford it, she stated she was able to budget for it:

I paid for it...it wasn't [hard]...in fact I even got money back...for one of them [births] cause we overpaid...I don't even remember what we paid. We just decided to have the baby and we paid it. (13).

Similarly, one participant felt that her out-of-pocket costs for childbirth were reasonable at the time, explaining:

No [didn’t previously have health insurance], but it, but it was cheap. It wasn't it - now I said cheap, but...it was still, you know, you have to pay and then we paid so much and then I would pay him monthly...everything might have been like maybe a thousand or something like that. You know, both the doctor and the hospital (17).

As these participants demonstrate, women without health insurance coverage during pregnancy were required to pre-pay for anticipated medical costs associated with delivery in order to access the local hospital.

### Access to Public Health Insurance Coverage

For low-income tribal members, public health insurance coverage was a vital resource that enabled them to access medical care. Many participants described their experiences with Medicaid and Medicare coverage, which provided many needed benefits but also had limitations. Many participants described how they used multiple forms of insurance to ensure adequate coverage, especially during pregnancy and youth. One participant reported qualifying for Medicaid for her pregnancy because of her low income:

Because of the way our income falls...I was able to qualify for Medicaid for all of my pregnancies....I have my own health insurance through my employer...but when I would get pregnant I also qualified for Medicaid…and they give you…so many months afterwards...So that was always covered (11).

This participant was able to use Medicaid as supplemental insurance to offset costs associated with pregnancy, but she notes that this coverage only extended a few months after the pregnancy ended. Another participant felt that without Medicaid she wouldn’t have been able to pay for all the healthcare she needed while pregnant: “I was able to access, I had good health care. I had great, I qualified for Medicaid….I went to all my prenatal visits…I had it good (29).”

One participant discussed the need for tribal members to learn about Medicaid enrollment to increase low-income people’s ability to access healthcare:

Getting the information out to our tribal members that they were able to come, that it was free of charge, scheduling appointments and different things like that that we were able to do. We were supposed to have some type of training for Medicaid, like for them to enroll in Medicaid and things like that. But that kind of never happened... So I think that's a big need....Lots and lots of people around this area do not have insurance....Majority of the people in these areas, the only insurance they have is if they have a really good job and they have insurance. Through their company....So like even my sister, she's a beautician so she doesn't have insurance. Because it would cost way too much. I know before I got this job with the tribe, I was paying like $350 a month and that was just for me. And the insurance was like even, nothing. People around here just can't afford that. Especially since they having kids that, you know, at 18, 19, 20 years old that’s young. And then having all of that money burdens, financial burdens, and then having to pay insurance on top of that (25).

As this participant emphasized, increasing awareness about and access to Medicaid coverage might go a long way in helping people to access healthcare who might not otherwise be able to afford it.

For young adults, Medicaid was often accessed through their parents’ coverage. One participant reported being covered both through her parent’s insurance, and through Medicaid, to cover what wasn’t through her primary insurance: “I have Blue Cross/Blue Shield and a secondary insurance, is it’s, Medicaid it's just to cover… My primary is with my dad, through his insurance and the other one is just me (14).” Another participant also reported being covered through her parent’s insurance: “Her [mother’s] insurance (18)” One participant expressed concern that younger tribal members were especially vulnerable to being without insurance coverage, once they aged off their parent’s insurance. Older folks were able to use Medicaid as a supplement to Medicare, but young adults were often left without access to coverage: “I think it would be the youngest one that wouldn't but the oldest got it all (4).” Another participant also reported relying on Medicaid for her pregnancies, but currently having private insurance through her employer. However, she expressed concern that her eldest daughter would be uninsured because she had aged out of the eligibility requirements that previously allowed her to be covered under her mother’s Medicaid: “No insurance….I had Medicaid to pay for everything because you know…[now] I have insurance through work….my daughter was…on Medicaid because I was [a] single mom, dad was diagnosed with cancer, so she was on Medicaid. Now that she's no longer…they told me she aged out (3)” These participants identify a time in life – young adulthood – when tribal members are potentially more vulnerable to lack of insurance coverage.

Older participants were sometimes able to combine Medicaid and Medicare to increase their coverage and improve their access to care, however, that wasn’t always enough. One participant stated that she qualified for both Medicaid and Medicare, but that some of her costs still came out of pocket:

Medicaid…Some of it's got to come out...from my pocket and some of them just takes it out of Medicare...Medicare takes care of your hospital and Medicaid takes care of your medicine (4).

Another participant described the ease of accessing care when covered by both Medicare and Medicaid: “And I have Medicare, and I have Medicaid. I can go anywhere…they're good with me (21).” However, she went on to state that because health insurance was so expensive, or didn’t adequately cover certain procedures or treatments, many tribal members had to wait until they qualified for Medicare to get treatment:

I think like the heart doctors, they're very, very expensive. People have to wait until Medicare before they can really get good care...instead of going to just anybody. You know, going to a specialist, heart specialist, and stuff. And once they get on Medicare, then they go straight to the heart doctor because now they can go, you know, the insurance is going to pay...but they can't go until they get on Medicare (21).

While many tribal members described their use of Medicaid and Medicare and the associated benefits of having such coverage, there remain significant limitations when it comes to who is eligible for these programs, the types of care they receive, and the amount and length of coverage.

## Discussion

These findings highlight the important role that insurance status played in participants ability to seek and pay for healthcare services. When juggling other financial responsibilities, healthcare needs were not always met. Participants described not having insurance as a significant barrier that kept them, and other tribal members from pursuing treatments, and paying for medications. Younger tribal members in particular described not having health insurance as a barrier that kept them from seeking healthcare services. These gaps in insurance coverage are concerning since American Indians experience extensive health disparities compared to the general population [1–3].

Although this topic has not been researched among state-recognized tribes, national literature has found that American Indian patients frequently make healthcare decisions based on their perceived ability to pay for services, and not based on doctor advice, or their own desire to seek care [16]. A lack of federal recognition comes with associated health and economic impacts related to the resources and increased political autonomy that result from federal recognition. The process for receiving federal recognition has received numerous critiques. It is a highly time-consuming and expensive process, in addition to being highly politicized and having inconsistently applied guidelines [4–7]. Federally-recognized tribal members can access health services for free via the IHS, but this important resource is not available to state-recognized tribes, further exacerbating health disparities for these tribal members. In addition, a lack of federal recognition means that members of this tribe do not have access to other resources available to federally-recognized tribes, such as monetary funds for programming, or community health centers. This is particularly important in relation to the finding that health literacy programs about insurance options and how to enroll in insurance is an important need identified in this study.

Although only 6% of the participants in this study reported being currently uninsured, which is below national levels of American Indian groups (15%), concerns about the ability to pay for healthcare were prominent among participants. Additionally, many participants recounted experiences of being uninsured at some point. Similar to national level findings, young and older tribal members were especially likely to be uninsured [19]. Access to public health insurance coverage was described as being important for all tribal members. States which have implemented the ACA Medicaid expansion have seen improvements in health insurance coverage for American Indian individuals [12]. Importantly, the most dramatic increases in coverage were seen for individuals who do not receive services through IHS. Frean et al. (2006) [12] also suggest that Medicaid expansion seems to be bringing in additional services to IHS, since IHS facilities can also be reimbursed by Medicaid. However, more research is needed to explore differences among specific regions and tribes.

Elder tribal members in this study described pre-paying for childbirth when uninsured, which sometimes acted as a barrier and restricted the types of care and facilities they could use. However, concerns about paying for childbirth were also reported by younger participants, emphasizing the need for increased health literacy for women who may be unaware of how to sign up for assistance that is available during the pregnancy and post-partum period. Coverage and ability to pay for reproductive and sexual health services is particularly essential for women of childbearing age, since 7 out of 10 women each year make at least once visit to a medical provider for these services [28]. Women with private health insurance are also more likely to receive contraceptive health insurance, compared to those paying for contraceptive services with Medicaid [28]. This discrepancy is concerning, since those covered by Medicaid should be eligible for the same type of care as those paying with private insurance. This suggests there may be additional barriers experienced by women that may need to be addressed. Expansion of Medicaid coverage may be one way to address this gap, though other structural changes are needed as well to ensure women, particularly women of color, receive high quality maternal healthcare.

Nationally, Medicaid covers over 25 million women [29]. This coverage was expanded in 2014 under the Affordable Care Act. Single mothers, women of color, and those with less formal education are most likely to be covered by Medicaid, providing up to 67% of women of reproductive age with family planning and other sexual and reproductive health services [29]. This is an essential source of healthcare for women during their reproductive years, as Medicaid acts as the largest source of payment for pregnancy and childbirth related services [29]. States that participated in the ACA’s Medicaid expansion saw improvements in maternal and infant mortality rates, in addition to decreases in the number of individuals who were uninsured while pregnant and in the postpartum period compared to states that did not expand Medicaid [30–32]. These findings indicate that increasing access to health insurance may play an important role in addressing some of the health disparities experienced by members of this tribe. Kozhimannil et al. [21] has argued that closing the insurance gap for American Indian women is one of the most important things we can do for addressing health disparities in this group. Many of the barriers highlighted by women can be addressed through healthcare infrastructure changes, such as extending post-partum Medicaid coverage from 60 days to one year, increasing available hospitals and specialists, improving access to contraceptive options, and recognizing tribal sovereignty in providing for healthcare.

Participants also described the need for education about insurance options and enrollment procedures for tribal members. Culturally relevant and appropriate health literacy education is an important area where future research is needed, as this topic has been under-studied for American Indian groups [19] and to our knowledge insurance health literacy interventions have not been studied for American Indian groups. Previous research has indicated that insurance status accounts for much of the variation in racial disparities in healthcare access, in particular in ensuring individuals have access to a regular healthcare provider [33]. However, research with American Indian individuals is limited, and caution should also be utilized to not over-state the importance of insurance coverage, since even when insurance status and other factors are similar, American Indian groups may still continue to experience health disparities because of a variety of additional factors such as discrimination, poverty, and increased rates of chronic and acute diseases [34].

Some important limitations of this study are that the findings are not intended to be generalizable. There is immense diversity among American Indian tribes in the United States and these results may not be applicable or relevant for other tribal nations. Women were only interviewed once, and future researchers may want to conduct multiple interviews to assess potential changes over time in insurance status. Future studies may also want to interview healthcare providers to explore their perception of the role of insurance status on healthcare access and patient outcomes. Incorporating quantitative data to assess insurance status among American Indian people is another important area for future investigation, particularly for those tribes who are not federally recognized. In addition, the first author conducted all interviews and completed the initial round of data analysis. This may have potentially biased our findings.

## Conclusion

This research begins to fill an important gap in the literature about the experiences of state-recognized tribal members in navigating the Western healthcare system and paying for healthcare. It sheds light on the role that being un- or under-insured plays in accessing healthcare, an area that has yet to be explored for non-federally recognized tribes. Of particular concern is the finding that being uninsured or under-insured kept tribal members from seeking healthcare and from filling needed prescriptions. Tribal members described having to pre-pay for healthcare when they were pregnant, and the need for increased coverage during that time period was discussed by women of all ages. Increased education about insurance options, how to enroll, and Medicaid expansion are potential approaches to begin to address these gaps in healthcare coverage. These findings also highlight the importance of increased research into this under-studied area, in addition to the need to promote tribal sovereignty and increased access to resources for non-federally recognized tribes, who do not receive healthcare services through IHS, as a way to combat current health disparities.

## Data Availability

The datasets generated and/or analyzed during the current study are not publicly available due to tribal concerns about privacy and confidentiality.

## Abbreviations

HIS: Indian Health Service
ACA: Affordable Care Act
CAB: Community Advisory Board
IRB: Institutional Review Board
PCP: Primary Care Physician
GED: General Educational Development
